# Immunization with Inactivated *Bacillus subtilis* Spores Expressing TonB-Dependent Receptor (TBDR) Protects Against Multidrug-Resistant *Acinetobacter baumannii* Infection

**DOI:** 10.3390/vaccines13060616

**Published:** 2025-06-06

**Authors:** Amalia A. Saperi, Atiqah Hazan, Nurfatihah Zulkifli, Hai-Yen Lee, Nor-Aziyah MatRahim, Sazaly AbuBakar

**Affiliations:** 1 Tropical Infectious Diseases Research and Education Centre (TIDREC), University Malaya, Kuala Lumpur 50603, Malaysia; aasaperi@um.edu.my (A.A.S.); atiqahazan@um.edu.my (A.H.); fatihahzulkifli@um.edu.my (N.Z.); leehaiyen@um.edu.my (H.-Y.L.); 2 Institute for Medical Research (IMR), Setia Alam, Shah Alam 40170, Malaysia; noraziyah@moh.gov.my

**Keywords:** *Acinetobacter baumannii*, *Bacillus subtilis*, pneumonia, spores, vaccine

## Abstract

Background/Objectives: The emergence of multidrug-resistant *Acinetobacter baumannii* (MDR *A. baumannii*) as a leading cause of fatal hospital-acquired infections underscores the urgent need for effective vaccines. While oral vaccines using live *Bacillus subtilis* spores expressing *A. baumannii* TonB-dependent receptor (TBDR) show promise, biosafety concerns regarding recombinant spore persistence necessitate alternative strategies. Here, we evaluated chemically inactivated *B. subtilis* spores displaying TBDR as a safer yet immunogenic vaccine candidate. Methods: Recombinant spores were inactivated using iron-ethanol sporicidal solution and administered to BALB/c mice (8–12 weeks old) to assess safety and immunogenicity. Toxicity was evaluated through clinical monitoring, serum biochemistry, and histopathology. Immune responses were characterized by T/B cell activation, IgG/IgA titers, and mucosal sIgA levels. Protective efficacy was determined by challenging immunized mice with MDR *A. baumannii* Ab35 and quantifying bacterial loads and examining tissue pathology. Results: The inactivated spores exhibited an excellent safety profile, with no adverse effects on clinical parameters, organ function, or tissue integrity. Immunization induced robust systemic and mucosal immunity, evidenced by elevated CD4+/CD8+ T cells, B cells, and antigen-specific IgG/IgA in serum and mucosal secretions. Following the challenge, vaccinated mice showed significantly reduced pulmonary bacterial burdens (>90% reduction), and preserved lung and spleen architecture compared to controls, which developed severe inflammation and tissue damage. Conclusions: These findings demonstrate that inactivated *B. subtilis* spores expressing TBDR are a safe, orally administrable vaccine platform that elicits protective immunity against MDR *A. baumannii*. By addressing biosafety concerns associated with live spores while maintaining efficacy, this approach represents a critical advance toward preventing high-risk nosocomial infections.

## 1. Introduction

Multidrug-resistant *Acinetobacter baumannii* (MDR *A. baumannii*), a Gram-negative coccobacillus, is a leading cause of hospital-acquired infections (HAIs), particularly in intensive care units (ICUs). Infection with the bacteria contributes to high mortality rates owing to its extensive multidrug resistance (MDR) properties [1]. Clinical manifestations of MDR *A. baumannii* infections include pneumonia, bacteremia, urinary tract infections (UTIs), wound infections, and meningitis, with ventilator-associated pneumonia and bloodstream infections being the most prevalent [2,3]. The scarcity of effective antibiotics and the absence of licensed vaccines against bacterial infection exacerbate the challenge of managing *A. baumannii* infections, making it a critical public health threat, particularly in nosocomial and combat-related wound settings [4,5].

Current therapeutic strategies rely heavily on combination antibiotic regimens, yet these approaches are often inadequate to ensure complete recovery, particularly in patients with debilitating conditions [6]. Consequently, prophylactic interventions, such as vaccines, could represent a promising alternative to mitigate *A. baumannii* infections. Several vaccine candidates have been explored, including subunit vaccines (e.g., OmpA, Blp1, CarO), whole-cell inactivated or ghost vaccines, chimeric constructs (e.g., TolC-MrcB), and DNA-based vaccines (e.g., nlpA, ompA + pal) [7]. Despite these efforts, significant hurdles, such as antigenic variability, insufficient immunogenicity, and safety concerns, hinder the development of a broadly effective vaccine [6].

An emerging strategy involves the use of recombinant generally recognized as safe (GRAS) bacterial spores as vaccine delivery platforms. *Bacillus subtilis*, a well-characterized GRAS organism, offers several advantages, including a proven safety profile, genetic tractability for antigen expression, and intrinsic adjuvant properties [8,9]. *B. subtilis* recombinant spores are highly stable, capable of withstanding harsh conditions, and particularly suited for oral administration due to their ability to survive gastrointestinal transit while stimulating robust mucosal and systemic immunity [10,11]. Furthermore, GRAS spores enhance antigen immunogenicity by promoting dendritic cell activation, antibody production, and memory responses, making them an attractive platform for vaccine development [12,13].

Recent advances in reverse vaccinology have identified TonB-dependent receptors (TBDRs) as promising antigenic targets due to their critical role in nutrient acquisition and surface exposure [14]. A recombinant *B. subtilis* spore-based vaccine expressing *A. baumannii* TBDR has demonstrated efficacy in eliciting robust mucosal and systemic immune responses, including elevated secretory IgA and serum antibody levels [15]. However, the potential for prolonged shedding of live recombinant spores raises biosafety concerns. To circumvent this issue, chemical inactivation of spores while preserving antigen immunogenicity presents a viable strategy for safer vaccine delivery. In this study, we evaluate the safety and immunogenic potential of chemically inactivated recombinant *B. subtilis* spores expressing *A. baumannii* TBDR and assess their ability to confer protection against MDR *A. baumannii* infection. By leveraging the safety and immunostimulatory properties of GRAS spores while addressing biosafety limitations, this approach may advance the development of a viable prophylactic oral vaccine against MDR *A. baumannii*.

## 2. Materials and Methods

### 2.1. Preparation of B. subtilis Spore Expressing A. baumannii TBDR

Recombinant *B. subtilis* expressing *A. baumannii* TBDR was recovered from glycerol stock and inoculated on a 2XYT plate. The recombinant *B. subtilis* stock was produced in our laboratory as published previously by MatRahim et al., 2023 [15]. A single colony containing the plasmid of interest was verified and enriched to the required volume for use as a starter culture. The recombinant bacterial culture was induced to sporulate, and spores were harvested after 32 h. The spore culture was centrifuged at high speed (14,000× *g*), and the bulk spore material was incubated in a water bath at 90 °C for 30 min. Bacterial spore purification was performed using polyethylene glycol (PEG) and a two-phase potassium buffer, followed by chemical inactivation with the sporicidal made of iron chloride, EDTA-2Na, and 50% ethanol solution, at 37 °C in a shaker incubator for four days. The final inactivated recombinant *B. subtilis* spore product was freeze-dried with the excipient and reconstituted with the diluent when needed for oral administration in the murine model.

### 2.2. Toxicity Study of Inactivated B. subtilis Spores Expressing A. baumannii TBDR

Untreated or inactivated *B. subtilis* spores expressing *A. baumannii* TBDR (1 × 10^11^ CFU/mL) were administered via oral gavage to eight 8–12 weeks female BALB/c mice per group over three consecutive days (200 µL per mouse). Mice were observed for 14 days for changes in weight, clinical parameter scores, and food intake. Serum samples were collected on day 17 by retro-orbital bleeding. On day 49, for immunogenicity testing, *n* = 2/group were euthanized for organ (kidney, liver, lung, spleen, heart) collection. The organs were fixed in 10% formalin, embedded in paraffin, and processed for histological examination with Hematoxylin and Eosin (H&E) staining to assess toxicity.

### 2.3. Clinical Parameter Scoring

Mice were observed and given clinical parameter scores (total score of 17). Clinical parameters observed and their breakdown were as follows: (1) stool consistency: normal, soft, soft with blood (score: 0–2); (2) posture: normal to hunched (score: 0–2); (3) spontaneous behavior: normal to no activity without disturbing (score: 0–2); (4) provoked behavior: normal to no activity after disturbing (score: 0–2); (5) evaluation of the eyes: clearness, openness (score: 0–3); (6) evaluation of the fur: cleanliness, gloss, smoothness (score: 0–3); (7) general appearance: undisturbed, mildly, moderately, severely disturbed (score: 0–3).

### 2.4. Immunogenicity of Inactivated B. subtilis Spores Expressing A. baumannii TBDR

The recombinant *B. subtilis* spores were administered orally according to the following groups (*n* = 8, 8–12 weeks female BALB/c mice/group): diluent control (group 1), *B. subtilis* spore control (group 2), 1 × 10^11^ CFU/mL untreated *B. subtilis* spores expressing *A. baumannii* TBDR (group 3), and 1 × 10^11^ CFU/mL inactivated *B. subtilis* spores expressing *A. baumannii* TBDR (group 4), with a total of three doses administered. Each dose consisted of three inoculations given on consecutive days (dose 1: days 1–3, dose 2: days 17–19, dose 3: days 33–35). Mice were monitored daily for 7 days after each dose for weight, clinical scores, and food intake. Blood samples (1% of average mouse weight) were collected through retro-orbital injection at every first inoculation of each dose (days 1, 17, and 33) and at the study’s conclusion (day 49) after anesthetization with ketamine/xylazine (80 mg/kg ketamine and 10 mg/kg xylazine). Fecal pellets were collected after a 4-h fasting period. Serum and fecal samples were collected from all mice. On day 49, two mice were euthanized with an overdose of ketamine/xylazine, and blood was drawn via cardiac puncture. Following this, bronchoalveolar lavage (BAL) fluid samples were taken and spleens were collected (*n* = 2 mice/group), and single-cell suspensions of splenocytes were isolated and stored in protein-free cryomedium synth-a-freeze (Gibco, Waltham, MA, USA) at −80 °C until needed.

### 2.5. Fluorescent Associated Cell Sorting (FACS)

Splenocytes were recovered from the freezer, washed, and resuspended in the stain buffer (BD Biosciences, Inc., Franklin Lakes, NJ, USA), adjusting the concentration to a minimum of 1 × 10^6^ cells/mL. Since we needed a high concentration of splenocytes, we pooled the 2 splenocytes collected per group as one sample. Aliquots of 100 µL were prepared in separate tubes. The Fc region was blocked with purified rat anti-mouse CD16/CD32 Mouse BD Fc Block (BD Biosciences, Inc., Franklin Lakes, NJ, USA). FITC Rat Anti-Mouse CD8a, PE Rat Anti-Mouse CD45R/B220, and APC-Cy7 Rat Anti-Mouse CD4 antibodies were added for staining. Prior to analysis, 7-AAD was added and incubated in the dark for 10 min. The cells were then analyzed using the FACS Canto II flow cytometer and BD FACSDiva™ Software Version 6.1.2 (BD Biosciences, Inc., Franklin Lakes, NJ, USA).

### 2.6. Enzyme-Linked Immunosorbent Assay (ELISA)

Flat-bottom 96-well polystyrene plates (Nunc MaxiSorp plates; Thermo Fisher Scientific, Waltham, MA, USA) were coated with inactivated *B. subtilis* spores expressing TBDR protein in 1× coating solution (KPL, SeraCare, Milford, MA, USA) at a concentration of 5 × 10^5^ CFU/well. This was performed through passive absorption overnight at 4 °C, followed by fixation with 10% formalin at 37 °C for 30 min and washing with the wash buffer provided by the ELISA kits using a plate washer (ThermoFisher, Waltham, MA, USA) three times. After washing, the wells were blocked with 1% BSA (SeraCare, Milford, MA, USA) in PBST for 2 h at room temperature, followed by another three washes with PBST. Subsequently, the samples (serum at 1:100, BAL at 1:20, or feces neat) diluted in PBST with 0.1% BSA were added to duplicate wells. The dilutions of the samples were optimized for the respective ELISA kits. The plates were then incubated overnight at 4 °C. Following incubation, the plates were processed strictly following instructions as provided by the ELISA kit manufacturer (Bio-techne, Minneapolis, MN, USA). The optical density (OD) values at 450 nm with 620 nm as the reference wavelength were recorded using a plate reader (Tecan Instruments, Mannedorf, Switzerland). The results were baseline-corrected against the control (diluent) group. This normalization was performed using GraphPad Prism version 9.0.0 ( GraphPad Software Inc., San Diego, CA, USA) after calculating the mean and SEM from *n* = 7/group (serum), *n* = 6/group (feces), or *n* = 2/group for BAL fluid.

### 2.7. Challenge of BALB/c Mice with A. baumannii Ab35

*A. baumannii* clinical isolate Ab35 was used for the study. The isolate was initially isolated from bronchoalveolar lavage (BAL) and made available for the study as a gift by Associate Professor Dr. Cindy Teh Shuan Ju, Universiti Malaya. Collection and use of the isolate obtained IRB approval [MEC no. 1073.21]. The isolate was recovered in Luria-Bertani (LB) broth containing 16 μg/mL of carbapenem (imipenem; Sigma-Aldrich, Burlington, MA, USA) to select for the multidrug-resistant (MDR) *A. baumannii*. BALB/c mice previously inoculated with the full dose of diluent control, *B. subtilis* spore control, untreated or inactivated recombinant *B. subtilis* spores (*n* = 5/group) were immunocompromised by inducing neutropenia through the administration of 1% cyclophosphamide monohydrate ( Sigma Aldrich, Burlington, MA, USA) with a total dose of 250 mg/kg by two intraperitoneal (IP) injections scheduled at day-4 (150 mg/kg) and day-1 (100 mg/kg) before the challenge. Then, 1 × 10^10^ CFU/mL of MDR *A. baumannii* (200 μL) was administered through oropharyngeal aspiration. The mice were observed for weight changes (up to day 14), clinical scores (up to day 5), and the amount of pellet consumed (weight of pellet) up to day 11.

### 2.8. Post-Challenge Lung Infection Histology and Burden Determination

Twenty-four hours after the challenge, mice (*n* = 2/group) were sacrificed by ketamine (80 mg/kg) and xylazine (10 mg/kg) overdose (0.1 mL/g), and their lungs were collected for histopathology. Lungs were removed, fixed in formalin, embedded in paraffin blocks, and stained with Hematoxylin and Eosin (H&E). All histology slides were scanned using a slide scanner (Pannoramic MIDI II; 3DHISTECH, Budapest, Hungary). For the subsequent study, 24 h after lung challenge, mice (*n* = 2/group) were sacrificed by ketamine-xylazine overdose, and their lungs were collected for bacterial lung burden assessment. The lungs were homogenized, plated on TSA plates, and incubated overnight at 37 °C. Bacterial count (CFU/mL) was observed and determined the following day.

### 2.9. Statistical Analysis

Data were plotted and analyzed using GraphPad Prism version 9.0.0 ( GraphPad Software Inc., San Diego, CA, USA) and are presented as means and standard error of the mean (SEM). Statistical analysis was performed to identify the normality of data using the Shapiro–Wilk test. The significance of the data was determined using the ordinary one way ANOVA and Dunnet’s multiple comparisons test. Differences were considered significant when *p* < 0.05.

## 3. Results

### 3.1. Toxicity of Inactivated B. subtilis Spores Expressing A. baumannii TBDR

The toxicity of inactivated *B. subtilis* spores expressing *A. baumannii* TBDR was assessed in BALB/c mice. Mice were inoculated with controls, untreated recombinant spores, or inactivated recombinant spores over three consecutive days and monitored for weight changes (days 1–14), clinical symptoms (days 1–5), and food consumption (days 1–11).

**Figure 1:**
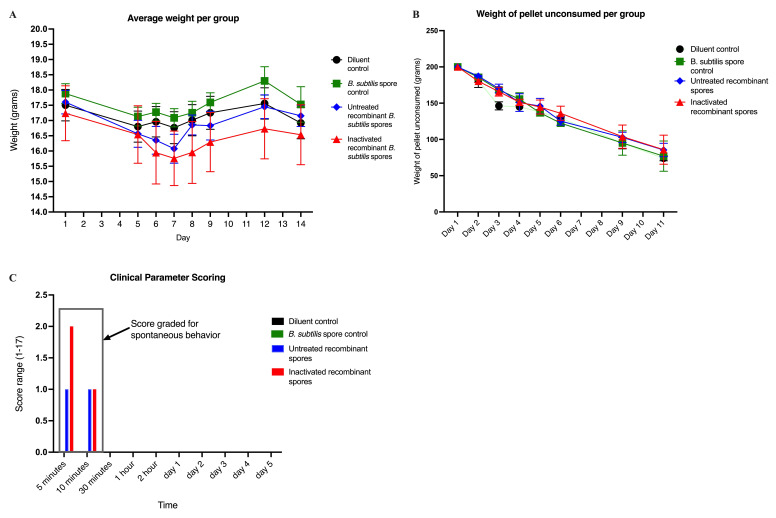
Toxicity testing in mice inoculated with *B. subtilis* spore expressing *A. baumannii* TBDR. (**A**) Average weight change of BALB/c mice (*n* = 10/group) from day 1 to day 14 following the first dose of diluent control, *B. subtilis* spore control, untreated or inactivated *B. subtilis* spores expressing *A. baumannii* TBDR (acute toxicity study). (**B**) The average weight of unconsumed pellets per group from day 1 to day 11 following the first dose of *B. subtilis* spores expressing *A. baumannii* TBDR protein or control groups. (**C**) Clinical parameter scoring of mice per group from day 1 to day 5 following the first dose of *B. subtilis* spores expressing *A. baumannii* TBDR protein or control groups.

Average starting weights on day 1 were 17.50 g (diluent control), 17.88 g (*B. subtilis* spore control), 17.60 g (untreated recombinant spores), and 17.24 g (inactivated recombinant spores). Weight fluctuations were observed over 14 days, but no significant differences were found between groups (*p* > 0.05) (Figure 1A). These results suggested that the administration of *B. subtilis* spores, whether untreated or inactivated, did not adversely affect the overall health or growth of the mice, as indicated by stable and comparable weight changes trending across all treatment groups.

Clinical symptoms were scored based on observations of behavior, posture, stool, and fur appearance. Mice treated with inactivated recombinant spores exhibited transient symptoms, with a maximum score of 2/17 (graded for spontaneous behavior) within the first 5 min post-inoculation. Scores decreased to 1/17 after 5 min, and no abnormalities were observed thereafter. For untreated recombinant spores, the maximum score was 1/17 during the first 10 min, with no abnormalities observed thereafter (Figure 1B). These results suggest that the administration of *B. subtilis* spores, whether untreated or inactivated, induced only mild and transient clinical symptoms, which resolved quickly without any lasting adverse effects.

Mice food consumption was measured by recording unconsumed pellet weights. All groups started with 200 g of pellets on day 1. By day 11, average unconsumed weights were 73.98 g (diluent control), 76.96 g (*B. subtilis* spore control), 85.44 g (untreated recombinant spores), and 85.87 g (inactivated recombinant spores). No significant differences were observed between groups (*p* > 0.05) (Figure 1C). These results suggested that the administration of *B. subtilis* spores, whether untreated or inactivated, did not significantly affect food consumption, indicating no adverse effects on appetite or feeding behavior.

Serum albumin and ALT levels were measured to assess the potential acute toxicity of the *B. subtilis* spore expressing *A. baumannii* TBDR protein. Average serum albumin levels were below the normal range (27–49 g/L) for all groups: 13 g/L (diluent control), 13 g/L (*B. subtilis* spore control), 14 g/L (untreated recombinant spores), and 11 g/L (inactivated recombinant spores). ALT levels were within the normal range (25–60 U/L): 59 U/L (diluent control), 37 U/L (recombinant spore control), 54 U/L (untreated recombinant spores), and 29 U/L (inactivated recombinant spores) (Table 1). These findings suggested that while serum albumin levels were low across all groups, the normal ALT levels suggested no significant liver damage or acute toxicity associated with the treatment.

**Figure 2:**
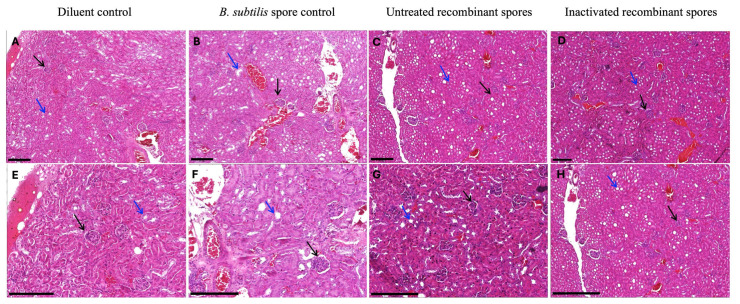
Kidney histology of female BALB/c mice inoculated with *B. subtilis* spores expressing *A. baumannii* TBDR. The figure shows the kidney H&E-stained histology of mice inoculated with either the diluent control, *B. subtilis* spore control, untreated recombinant spores or inactivated recombinant spores. Figure (**A**–**D**) represent the kidney histology at 10× magnification, and figure (**E**–**H**) represents the kidney histology at 20× magnification. Arrows indicate the glomerulus, and arrows in blue indicate the tubules. Scale bar presented at 0.2 mm.

**Figure 3:**
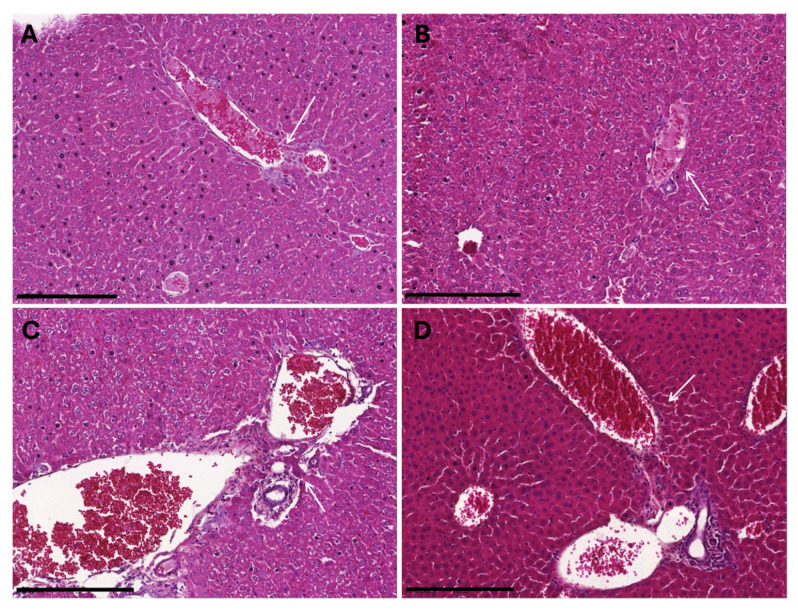
Liver histology of female BALB/c mice inoculated with *B. subtilis* spores expressing *A. baumannii* TBDR. The figure shows the liver histology of mice inoculated with (**A**) diluent control, (**B**) *B. subtilis* spore control, (**C**) untreated recombinant spores or, (**D**) inactivated recombinant spores. Liver histology presented at 20× magnification. The arrow in white indicates the portal tract. The scale bar is presented at 0.2 mm.

No histological abnormalities of mice kidneys and livers (*n* = 2/group) were observed. Kidneys were normal, including well-vacuolated tubules and normal glomeruli (Figure 2). Livers exhibited flat epithelium, no endothelial vacuolation, and no increase in edema or connective tissue (Figure 3). These results suggest that the administration of *B. subtilis* spores, whether untreated or inactivated, did not induce any pathological changes in the kidneys or livers of mice, further supporting the safety profile of the treatment.

Results from the toxicity assessment studies suggested that *B. subtilis* spores expressing *A. baumannii* TBDR did not significantly affect weight, clinical symptoms, and food consumption or induce acute toxicity, as evidenced by normal ALT levels and the absence of histological abnormalities in the kidneys and livers of inoculated mice. Overall, these results support the safety and tolerability of the recombinant bacterial spores.

### 3.2. Immunogenicity of Inactivated B. subtilis Spores Expressing A. baumannii TBDR

**Figure 4:**
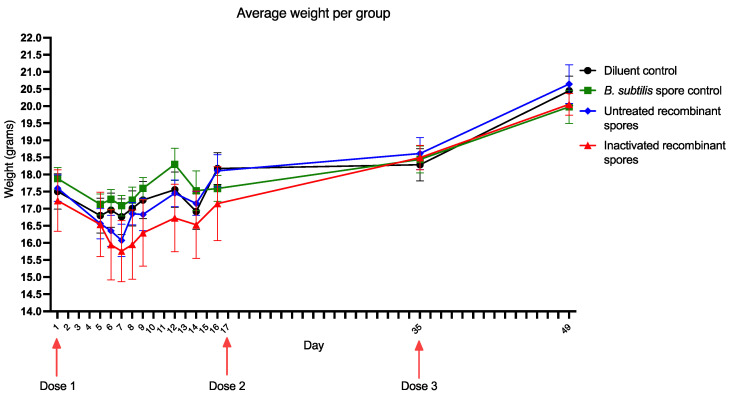
The average weight of mice administered *B. subtilis* spores expressing *A. baumannii TBDR.* Average weight change of BALB/c mice per group (*n* = 8/group) administered diluent control, *B. subtilis* spore control, untreated, or inactivated *B. subtilis* spores expressing *A. baumannii* TBDR. Weights were recorded on day 1 (first dose), day 17 (second dose), and day 35 (third dose), with observations continuing until day 49 (evaluation of immunogenicity).

The immunogenicity of inactivated *B. subtilis* spores expressing *A. baumannii* TBDR was evaluated in BALB/c mice. Mice were administered three doses of either the diluent control, *B. subtilis* spore control, untreated, or inactivated recombinant *B. subtilis* spores at varying concentrations. Mice’s body weights were recorded before each dose and at the endpoint (day 49). Average starting weights on day 1 were 17.50 g (diluent control), 17.88 g (*B. subtilis* spore control), 17.60 g (untreated recombinant spores), and 17.24 g (inactivated recombinant spores). By day 49, average weights were 20.45 g, 19.98 g, 20.65 g, and 20.05 g, respectively (Figure 4). The result indicated no significant changes between the treatment groups and the diluent control (*p* > 0.05).

**Figure 5:**
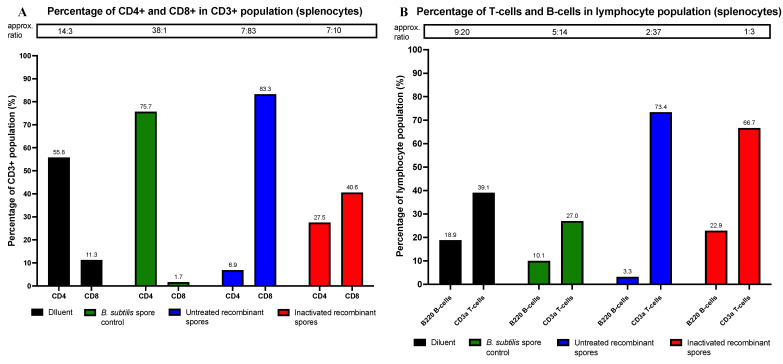
CD4+ versus CD8+ T-helper cells and CD3 T-cells versus B220 B-cells in mice inoculated with *B. subtilis* spore expressing *A. baumannii* TBDR. BALB/c mice were inoculated with diluent control, *B. subtilis* spore control, untreated, or inactivated *B. subtilis* spores expressing *A. baumannii* TBDR. On day 49 (endpoint for objective 3), two mice per group were sacrificed, and spleens were harvested. Splenocytes were isolated and analyzed using flow cytometry to determine the percentage of (**A**) CD4+ and CD8+ helper T-cells in the CD3+ population of splenocytes (**B**) CD3 T-cells and B220 B-cells in the splenocytes.

Spleens were harvested from *n* = 2 mice/group, 14 days after the final dose. Splenocytes were analyzed for CD4+ and CD8+ Helper T-cell, B220+ B-cell, and CD3+ T-cell populations using flow cytometry. For splenocytes, CD4+ and CD8+ percentages were 55.8% and 11.3% (diluent control), 75.7% and 1.7% (*B. subtilis* spore control), 6.9% and 83.3% (untreated recombinant spores), and 27.5% and 40.6% (inactivated recombinant spores) (Figure 5A). These results suggest that untreated recombinant spores induced a strong CD8+ T-cell response, while inactivated recombinant spores showed a balanced CD4+ and CD8+ T-cell profile.

Splenocytes were also analyzed for B220+ B-cells and CD3+ T-cells. Results were 18.9% and 39.1% (diluent control), 10.1% and 27.0% (*B. subtilis* spore control), 3.3% and 73.4% (untreated recombinant spores), and 22.9% and 66.7% (inactivated recombinant spores) (Figure 5B). These results suggest that untreated recombinant spores induced a strong CD3+ T-cell response, while inactivated spores showed a balanced B-cell and T-cell profile.

The humoral immune responses against the *B. subtilis* spores expressing *A. baumannii* TBDR were evaluated by determining the level of PHPS9-1PR82PT6XHis-specific IgG, IgA, and secretory IgA (sIgA) antibodies in serum, bronchoalveolar lavage (BAL) fluid, and fecal samples. Serum samples were collected 14 days after each dose, while BAL fluid and fecal samples were collected 14 days after the final dose. Antibody levels were quantified using ELISA.

**Figure 6:**
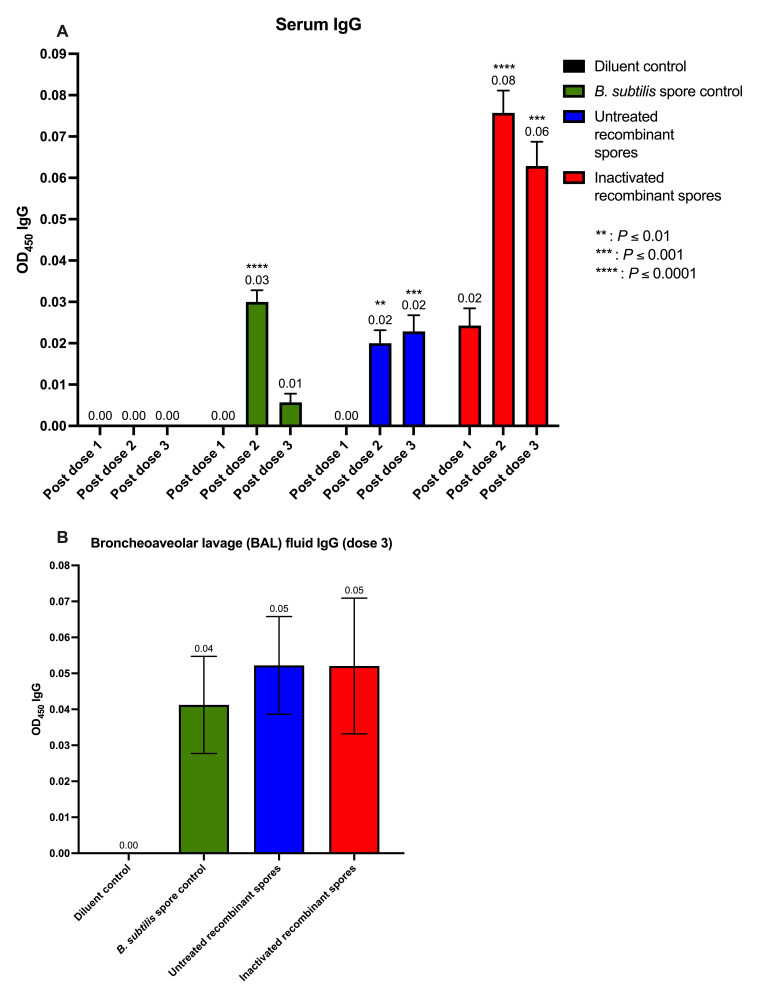
Detection of spore-specific IgG in serum and bronchoalveolar lavage (BAL) fluid of mice after inoculation with *B. subtilis* spores expressing *A. baumannii* TBDR. Serum from BALB/c mice (*n* = 7/group) were analyzed using ELISA to measure TBDR spore-specific IgG antibody measured by wavelength (OD450) following inoculation with diluent control, *B. subtilis* spore control, untreated or inactivated *B. subtilis* spores expressing *A. baumannii* TBDR protein. Measurements were taken 14 days after (**A**) doses 1, 2, and 3. (**B**) Mice BAL fluid (*n* = 2/group) collected at endpoint day 49 were also observed.

In serum, IgG levels (Figure 6A) were negligible after the first dose, except for inactivated *B. subtilis* spores expressing *A. baumannii* TBDR (0.02 nm, *p* < 0.0049). Following the second dose, IgG levels increased significantly, for untreated (0.02 nm, *p* = 0.0021) and inactivated recombinant spores (0.08 nm, *p* < 0.0001). This trend persisted after the third dose, with elevated IgG levels observed for untreated (0.02 nm, *p* = 0.0008) and inactivated recombinant spores (0.06 nm, *p* < 0.0001). BAL fluid IgG levels had a slight elevation in all groups compared to the diluent control, although not significant (*p* > 0.05) (Figure 6B). The results that were not baseline-corrected relative to the diluent group were plotted on a dot plot (Appendix A).

**Figure 7:**
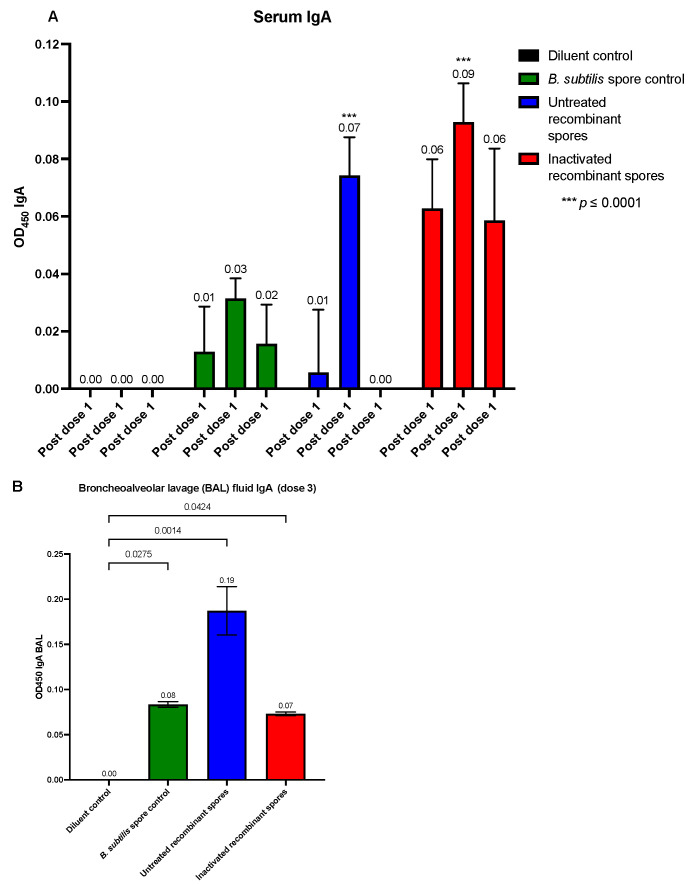
Detection of spore-specific IgA in serum and bronchoalveolar lavage (BAL) fluid of mice after inoculation with *B. subtilis* spores expressing *A. baumannii* TBDR. Serum from BALB/c mice (*n* = 7/group) were analyzed using ELISA to measure TBDR spore-specific IgA antibody measured by wavelength (OD450) following inoculation with diluent control, *B. subtilis* spore control, untreated or inactivated *B. subtilis* spores expressing *A. baumannii* TBDR. Measurements were taken 14 days after (**A**) doses 1, 2, and 3. (**B**) Mice BAL fluid (*n* = 2/group) collected at endpoint day 49 were also observed.

Serum IgA levels (Figure 7A) were minimal after the first dose, with a slight increase observed for inactivated *B. subtilis* spores expressing *A. baumannii* TBDR (0.06 nm). After the second dose, significant IgA responses were detected for untreated (0.07 nm, *p* = 0.0002) and inactivated recombinant spores (0.09 nm, *p* < 0.0001). Following the third dose, IgA levels were elevated for inactivated recombinant spores (0.06 nm), although the *p* value is not significant. In BAL fluid, IgA levels were highest for untreated recombinant spores (0.19 nm, *p* = 0.0014, while inactivated recombinant spores also had a significant increase (0.07 nm, *p* = 0.0424), suggesting a robust mucosal IgA response for both untreated and inactivated recombinant spores (Figure 7B). The results that were not baseline-corrected relative to the diluent group were plotted on a dot plot (Appendix B).

**Figure 8:**
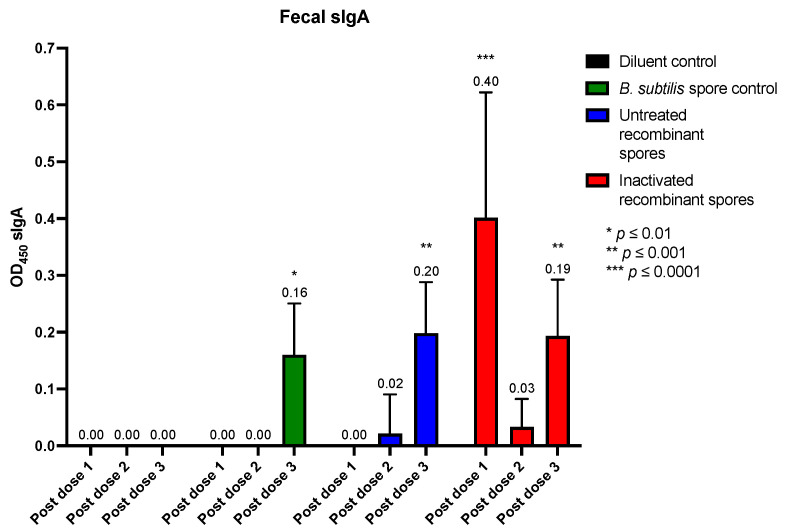
Detection of spore-specific secretory IgA (sIgA) in fecal samples of mice after inoculation with *B. subtilis* spores expressing *A. baumannii* TBDR. Fecal samples from BALB/c mice (*n* = 6/group) were analyzed using ELISA to measure TBDR spore-specific sIgA antibody measured by wavelength (OD450) following inoculation with diluent control, *B. subtilis* spore control, untreated or inactivated *B. subtilis* spores expressing *A. baumannii* TBDR. Measurements were taken 14 days after doses 1, 2 and 3.

Fecal sIgA levels were measured to assess intestinal immune responses (Figure 8). After the first dose, a significant sIgA response was observed for inactivated *B. subtilis* spores expressing *A. baumannii* TBDR (0.40 nm, *p* < 0.0001). After the second dose, sIgA levels were low across all groups. However, after the third dose, significant sIgA responses were detected for the *B. subtilis* spore control (0.16 nm, *p* = 0.0172), and inactivated recombinant spores at (0.19 nm, *p* = 0.0030). These findings suggested that inactivated spores can stimulate systemic (serum) as well as mucosal (BAL fluid and fecal) immune responses. The results that were not baseline-corrected relative to the diluent group were plotted on a dot plot (Appendix C).

### 3.3. Protective Efficacy of Inactivated B. subtilis Spores Expressing A. baumannii TBDR Against MDR A. baumannii Challenge

Following inoculation with the diluent control, *B. subtilis* spore control, untreated (or inactivated *B. subtilis* spores expressing *A. baumannii* TBDR, mice were challenged with 1 × 10^10^ CFU/mL MDR *A. baumannii* Ab35. Mice were monitored for weight changes (days 1 to 39), and clinical scores (days 1 to 13).

**Figure 9:**
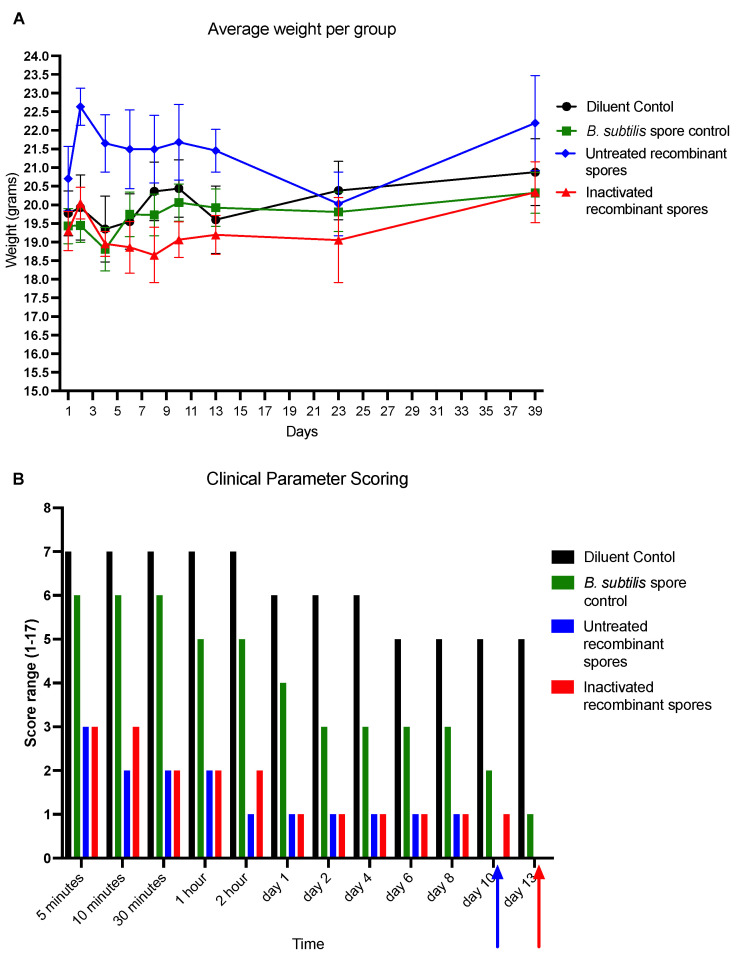
*MDR A. baumannii* challenge in BALB/c mice inoculated with *B. subtilis* spores expressing *A. baumannii* TBDR. (**A**) Average weight change of mice inoculated with diluent control, *B. subtilis* spore control, untreated or inactivated *B. subtilis* spores expressing *A. baumannii* TBDR, following challenge with MDR *A. baumannii* Ab35 up to day 39. (**B**) Average clinical parameter scoring per group from day 1 to day 13 post-infection with MDR *A. baumannii*.

Average starting weights on day 1 ranged from 19.30 g to 20.70 g across groups. By day 39, final weights ranged from 19.87 g to 22.20 g, with no significant differences between groups (*p* > 0.05) (Figure 9A). Clinical scores based on behavior, posture, stool, and fur appearance were recorded at intervals up to day 13 (Figure 9B). At 5 min, scores were 7 (diluent control), 6 (*B. subtilis* spore control), 3 (untreated recombinant spores 1 × 10^11^ CFU/mL), and 3 (inactivated recombinant spores 1 × 10^11^ CFU/mL) with higher scores indicating a more scrunched posture, disturbed spontaneous and provoked behavior, and irritation. Scores at subsequent time points showed a gradual decline in clinical scores across all groups. By day 13, scores were 5 (diluent control), 1 (*B. subtilis* spore control), 0 (untreated recombinant spores), and 0 (inactivated recombinant spores). These results showed groups of mice that received untreated recombinant spores or inactivated recombinant spores exhibited no clinical signs of illness by day 13 upon challenge with MDR *A. baumannii*, suggesting that groups that received *B. subtilis* spores, tolerated challenge with the MDR *A. baumannii* infection. No lethality in any group was observed.

**Figure 10:**
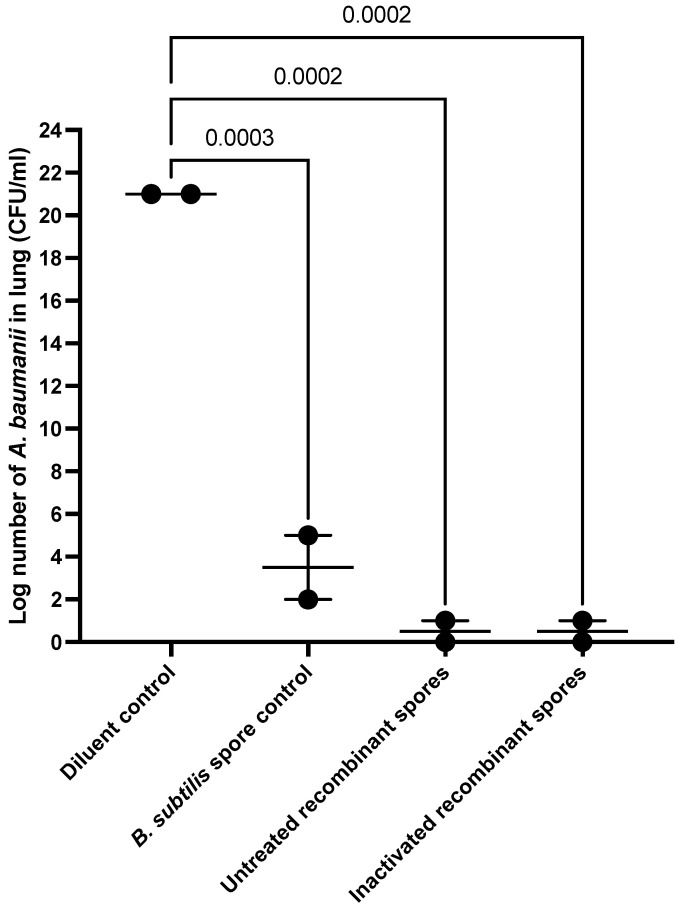
Lung homogenate of BALB/c mice inoculated with *B. subtilis* spores expressing *A. baumannii* TBDR and challenged with MDR *A. baumannii* Ab35. Lungs from mice inoculated with diluent control, *B. subtilis* spore control, untreated, or inactivated *B. subtilis* spores expressing *A. baumannii* TBDR were isolated 24 h after challenge with MDR *A. baumannii* Ab35. Lung homogenates were plated in duplicates on Tryptone Soy Agar (TSA) to quantify *A. baumannii* Ab35.

Lungs of mice from the groups given the *B. subtilis* spore control, untreated, or inactivated *B. subtilis* spores expressing *A. baumannii* TBDR were homogenized and plated 24 h post-challenge with MDR *A. baumannii* Ab35 to quantify bacterial burden (Figure 10). The lungs of challenged mice treated with the diluent control exhibited a bacterial load of 1.32 log_10_ CFU/mL (21 CFU/mL). In contrast, mice treated with untreated or inactivated recombinant *B. subtilis* spores showed significantly lower bacterial loads: 0.5 CFU/mL for untreated recombinant spores and 0.5 CFU/mL for inactivated recombinant spores, based on averaged duplicate plates. These results suggested that inoculation with the recombinant *B. subtilis* spores significantly reduced (*p* < 0.001) the bacterial burden in the lungs, demonstrating potential efficacy against MDR *A. baumannii* infection.

**Figure 11:**
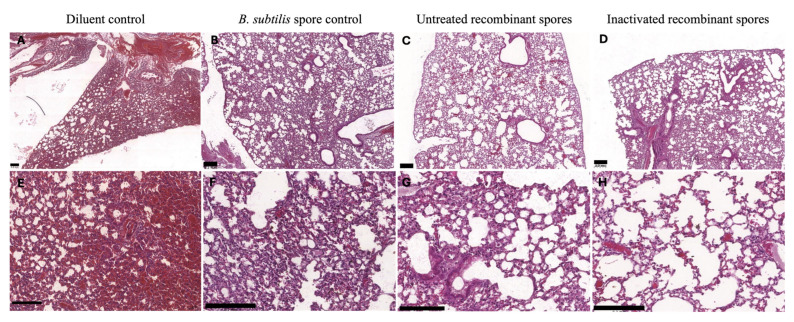
Lung histology of female BALB/c mice inoculated with *B. subtilis* spores expressing MDR *A. baumannii* TBDR and challenged with MDR *A. baumannii* Ab35. The figure shows lung histology of mice inoculated with either the diluent control, *B. subtilis* spore control, untreated recombinant spores or inactivated recombinant spores and challenged with MDR *A. baumannii* Ab35. Figure (**A**–**D**) represent the lung histology at 5× magnification, and figure (**E**–**H**) represents the lung histology at 20× magnification. The scale bar is presented at 0.2 mm.

The lung and spleen tissues from immunized mice were examined 24 h post-challenge with 1 × 10^10^ CFU/mL MDR *A. baumannii* Ab35. Lungs from mice immunized with the diluent control exhibited pathological characteristics, including thickened air-sac interstitium with uniform density and increased cellularity within the air sacs (Figure 11A). Tissues from the *B. subtilis* spore control group displayed slightly thicker, patchy air-sac interstitium with increased cellular content (Figure 11B). Tissues from mice treated with untreated recombinant spores (Figure 11C), and inactivated recombinant spores (Figure 11D) presented normal lung histology, with no increased cellularity and a slight presence of alveolar macrophages, indicative of an active immune response against infection.

**Figure 12:**
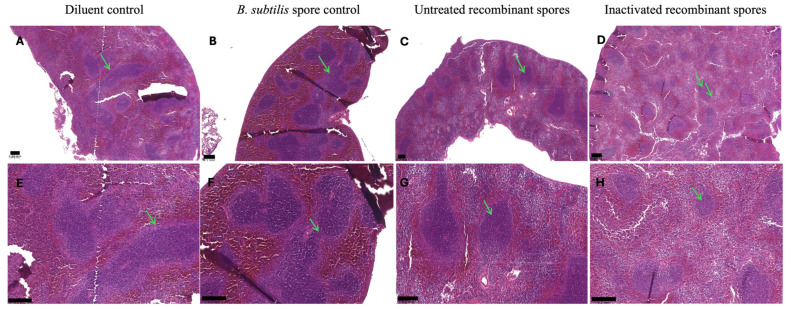
Spleen histology of female BALB/c mice inoculated with *B. subtilis* spores expressing MDR *A. baumannii* TBDR and challenged with MDR *A. baumannii* Ab35. The figure shows spleen histology of mice inoculated with either the diluent control, *B. subtilis* spore control, untreated recombinant spores or inactivated recombinant spores and challenged with MDR *A. baumannii* Ab35. Figure (**A**–**D**) represent the spleen histology at 4× magnification, and figure (**E**–**H**) represents the spleen histology at 10× magnification. Arrows in green indicate germinal centers. The scale bar is presented at 0.2 mm.

Spleens from mice immunized with the diluent control (Figure 12A) or *B. subtilis* spore control (Figure 12B) displayed enlarged germinal centers, suggesting heightened lymphoid cell and T-cell production. In contrast, tissues from mice treated with untreated recombinant spores (Figure 12C), or inactivated recombinant spores (Figure 12D) showed normal-sized germinal centers with no signs of enlargement. These findings suggest that untreated and inactivated *B. subtilis* spores, effectively mitigated pathological changes in the lungs and normalized immune activity in the spleen. Overall, these results demonstrated that treatment with inactivated *B. subtilis* spores expressing *A. baumannii* TBDR, was well-tolerated and effective in reducing bacterial burden and pathological changes in mice challenged with MDR *A. baumannii*.

## 4. Discussion

The rise of multidrug-resistant *Acinetobacter baumannii* (MDR *A. baumannii*) as a leading cause of nosocomial infections has created an urgent need for effective preventive strategies, particularly in intensive care settings where mortality rates remain alarmingly high. Our study investigated the potential of *Bacillus subtilis* spores expressing the TonB-dependent receptor (TBDR) of *A. baumannii* as an oral vaccine platform, comparing the safety and immunogenicity of live (untreated) versus chemically inactivated recombinant spores. The findings demonstrate that both formulations are well-tolerated and immunogenic, with inactivated spores offering distinct advantages in terms of balanced immune activation while addressing biosafety concerns associated with the use of live recombinant microorganisms.

Results from the present study demonstrate that both untreated and inactivated *Bacillus subtilis* spores expressing *A. baumannii* TBDR are safe and immunogenic, with inactivated spores offering particular advantages for clinical translation. Comprehensive toxicity assessments revealed no adverse effects on murine weight, organ function, or histopathology, consistent with our previous findings using live spores [15]. Over the 14-day observation period, no significant differences in weight changes were observed between mice treated with untreated recombinant spores, inactivated recombinant spores, or the control groups, indicating that the administration of the recombinant spores did not adversely affect overall health or growth. Similarly, clinical symptoms were mild and transient, resolving within minutes post-inoculation, which suggests that the spores did not induce any lasting adverse effects on behavior, posture, stool, or fur appearance. Food consumption patterns also remained consistent across all groups, further confirming that the treatment did not impair appetite or feeding behavior.

Biochemical profiling of the serum collected from mice immunized with untreated or inactivated *B. subtilis* spores expressing *A. baumannii* TBDR suggested that serum albumin levels, although below the normal range in the untreated and inactivated recombinant spore groups, were consistent with the control groups, suggesting that the low levels were not specifically related to the administration of recombinant spores. Importantly, ALT levels, a key marker of liver function, remained within the normal range for all groups, indicating no significant liver damage or acute toxicity associated with the treatment. Histological analysis of the kidneys and livers of mice given the untreated or inactivated recombinant spores, further strengthens these findings, as no abnormalities were observed in these organs compared to the control groups.

The immunogenicity of untreated and inactivated *B. subtilis* spores expressing *A. baumannii* TBDR revealed differences in how untreated versus inactivated spores stimulate host immunity. The data obtained from the study suggested that untreated recombinant spores, strongly favored a predominantly Th1-type response, characterized by robust CD8+ T cell activation, which aligns with their ability to persist and potentially undergo limited germination in the gastrointestinal tract. This persistence likely facilitates prolonged antigen presentation through MHC class I pathways, favoring cytotoxic T lymphocyte responses valuable for intracellular pathogen clearance [16]. The sustained presence of live spores may stimulate a more robust Th1 response, which is crucial for effective cell-mediated immunity (CMI) [17]. In contrast, inactivated spores suggested a more balanced Th1/Th2 profile with stronger CD4+ T cell and B cell activation. This balanced response is particularly advantageous for extracellular pathogens like *A. baumannii*, where both antibody-mediated neutralization and T-cell-mediated immunity could contribute to protection [18].

Previous studies by de Souza et al. (2014) showed that the administration of p24 (HIV protein) as a vaccine component resulted in a Th1-predominant response [15]. However, when p24 was co-administered with live and inactivated *B. subtilis* spores, the adjuvant properties of the spores elicited both Th1 and Th2 responses, as indicated by a balanced IgG2a to IgG1 ratio [12]. These observations were similar to those observed from the present study, where inactivated *B. subtilis* spores elicited a balanced Th1/Th2 response. A possible explanation for this balanced response is that the spores are efficiently facilitated through both the MHC class I and class II presentation pathways [19]. Additionally, spores may activate one or more pattern recognition receptors (PRRs) across various dendritic cell (DC) subsets [12,20], thereby modulating the immune response.

The evaluation of humoral immune responses against *B. subtilis* spores expressing *A. baumannii* TBDR demonstrated that both untreated or inactivated spores induce robust antibody production, with distinct patterns in systemic (serum) and mucosal (BAL fluid and fecal) immunity. Inactivated spores elicited earlier and stronger IgA responses in mucosal compartments, including the respiratory and intestinal tracts—critical barrier sites for *A. baumannii* colonization, highlighting the platform’s ability to rapidly stimulate mucosal immunity, a feature often lacking in parenteral vaccines [21]. Systemic IgG responses were enhanced with boosting, particularly for inactivated spores, suggesting their suitability for prime-boost regimens. The concurrent stimulation of mucosal and systemic immunity is crucial for protection against *A. baumannii*, which can cause both localized and disseminated infections. Robust IgG and IgA responses in serum, alongside elevated IgA in BAL and sIgA in feces, confirmed the ability of both spore formulations to effectively engage both immune compartments, aligning with earlier findings where live spores induced elevated serum IgG and fecal sIgA. While untreated spores favored a Th1-biased response, inactivated spores promoted a balanced Th1/Th2 response, yet both formulations outperformed the diluent control, underscoring their immunogenic potential. These results position both live and inactivated spores as promising vaccine candidates, with inactivated spores offering additional advantages due to their balanced immune activation and strong mucosal priming.

Both formulations also significantly reduced bacterial loads in challenged mice and prevented the severe lung pathology observed in controls, confirming the protective efficacy of TBDR as a target antigen. These findings are in concordance with earlier reports showing bacteriolytic activity in sera from immunized mice [15], while addressing the biosafety concerns of live recombinant microorganisms using chemically inactivated recombinant spores.

The challenge study undertaken here demonstrated the protective efficacy of both spore formulations against MDR *A. baumannii* infection. Immunized mice showed no clinical signs of illness by day 13 post-challenge, while control groups exhibited persistent symptoms. Notably, histopathological analysis revealed near-normal lung architecture in vaccinated mice, contrasting sharply with the pronounced interstitial thickening and cellular infiltration seen in controls. The preservation of normal germinal center size in the spleens of immunized mice further confirmed effective infection control. Together, these findings demonstrate that TBDR-expressing spores not only prevent bacterial colonization but also mitigate infection-induced tissue damage and inflammatory responses.

Several limitations of the current study warrant discussion. The unexpected immune stimulation by the diluent control complicated the interpretation of the immunogenicity data, underscoring the importance of including a different negative control in future studies. This response could have been due to the presence of inulin in the diluent formulation. Inulin, a naturally occurring polysaccharide composed of fructose units linked with a terminal glucose unit [22], was included for its adjuvant properties. Beyond its role as a dietary fiber and stabilizer, inulin has demonstrated potential as an immunomodulatory adjuvant [23], and can modulate the immune system by influencing the gut microbiota [24]. Inulin-based adjuvants, such as Advax, have been shown to enhance vaccine efficacy by promoting antigen uptake and activation of T and B cells, leading to a stronger and longer-lasting immune response [25,26,27]. While inulin’s role as an adjuvant is still under investigation, its immunomodulatory potential makes it a promising candidate for enhancing vaccine efficacy. To better isolate the immune response attributable to the spores, it would have been beneficial to include a PBS control group, which would have provided a clearer baseline for comparison by excluding the potential immune-stimulating effects of inulin. Despite this limitation, the immune response values observed in mice groups immunized with the untreated or inactivated recombinant spore groups were much higher compared to just the diluent control in most results. This suggests that the immune-stimulating effects of inulin, while present, did not overshadow the robust immune responses elicited by the recombinant spores.

Other limitations include technical challenges with splenocyte viability and the small sample sizes for certain assays reduced the statistical power of some immune analyses. In compliance with our institution’s Animal Ethics Committee guidelines, we adhered to strict animal use limits, balancing research requirements with ethical considerations to ensure minimal animal usage. Additionally, while the study demonstrated protection against acute infection, longer-term studies are needed to evaluate the durability of immune responses and protection. Future work should also explore whether the vaccine can prevent or ameliorate other manifestations of *A. baumannii* infection, such as bacteremia or wound infections.

The successful use of inactivated spores in this study addresses a major biosafety concern regarding the environmental release of live recombinant microorganisms while maintaining effective immunogenicity. This finding has important implications for vaccine development of the bacterial spore platform, suggesting that chemical inactivation can be employed without compromising protective efficacy. The approach may be particularly valuable for immunocompromised populations where live vaccines could be contraindicated. Furthermore, the bacterial spore platform’s flexibility for expressing different antigens positions it as a potential strategy for targeting other multidrug-resistant pathogens.

## 5. Conclusions

In summary, this study establishes inactivated *B. subtilis* spores expressing TBDR as a promising oral vaccine candidate against MDR *A. baumannii*. The platform’s excellent safety profile, ability to stimulate both mucosal and systemic immunity, and demonstrated efficacy in protection studies highlight its potential for clinical translation. The balanced immune response elicited by inactivated spores, combined with their practical advantages for manufacturing and distribution, make them particularly attractive for further development. Future studies should focus on optimizing immunization protocols, evaluating protection in additional infection models, and advancing the most promising formulations toward clinical evaluation.

## Figures and Tables

**Table 1:** Serum albumin and serum ALT of mice inoculated with *B. subtilis* spores expressing *A. baumannii* TBDR.

Test Groups				
	Diluent Control	*B. subtilis* Spore Control	Untreated *B. subtilis* Spores	Inactivated *B. subtilis* Spores
Albumin	13 g/L (low)	13 g/L (low)	14 g/L (low)	11 g/L (low)
ALT	59 U/L (normal)	37 U/L (normal)	54 U/L (normal)	29 U/L (normal)
**Normal mice reference**				
Albumin	27–49 g/L			
ALT	25–60 U/L			

## Data Availability

All data has been reported in the figures stated in the journal. The data that support the findings of this study are available from the corresponding author upon reasonable request.

## Appendix A

Detection of spore-specific IgG in serum and bronchoalveolar lavage (BAL) fluid of mice after inoculation with *B. subtilis* spores expressing *A. baumannii* TBDR (Original values, not baseline corrected). Serum from BALB/c mice (*n* = 7/group) were analyzed using ELISA to measure TBDR spore-specific IgG antibody measured by wavelength (OD450) following inoculation with diluent control, *B. subtilis* spore control, untreated or inactivated *B. subtilis* spores expressing *A. baumannii* TBDR protein. Measurements were taken 14 days after (**A**) dose 1 (**B**) dose 2 (**C**) and dose 3. (**D**) Mice BAL fluid (*n* = 2/group) collected at endpoint day 49 were also observed.

## Appendix B

Detection of spore-specific IgA in serum and bronchoalveolar lavage (BAL) fluid of mice after inoculation with *B. subtilis* spores expressing *A. baumannii* TBDR (Original values, not baseline corrected). Serum from BALB/c mice (*n* = 7/group) were analyzed using ELISA to measure TBDR spore-specific IgA antibody measured by wavelength (OD450) following inoculation with diluent control, *B. subtilis* spore control, untreated or inactivated *B. subtilis* spores expressing *A. baumannii* TBDR. Measurements were taken 14 days after (**A**) dose 1, (**B**) dose 2, and (**C**) dose 3. (**D**) Mice BAL fluid (*n* = 2/group) collected at endpoint day 49 were also observed.

## Appendix C

Detection of spore-specific secretory IgA (sIgA) in fecal samples of mice after inoculation with *B. subtilis* spores expressing *A. baumannii* TBDR. Fecal samples from BALB/c mice (*n* = 6/group) were analyzed using ELISA to measure TBDR spore-specific sIgA antibody measured by wavelength (OD450) following inoculation with diluent control, *B. subtilis* spore control, untreated or inactivated *B. subtilis* spores expressing *A. baumannii* TBDR. Measurements were taken 14 days after (**A**) dose 1, (**B**) dose 2, and (**C**) dose 3.
